# American Institute of Homeopathy

**Published:** 1897-04

**Authors:** Eugene H. Porter

**Affiliations:** New York City; General Secretary; Buffalo, New York


### AMERICAN INSTITUTE OF HOMEOPATHY.

New York City, March 24th, 1897.

EDITOR OF THE HOMEOPATHIC PHYSICIAN:—Before the American Institute of Homeopathy adjourned at Detroit last year it was agreed to make the coming session at Buffalo, '97, the greatest and most successful one in the history of the organization. This determination has not been lessened, and the efforts of the officers, and chairmen, and friends of the Institute have been loyally and enthusiastically seconded by the profession everywhere.

Centrally located, reached by all the great trunk lines of the East and West, connected by boat with all the lake ports, with splendid and ample accommodations for all who may come, Buffalo justly expects a host of homeopathic doctors next June.

The American Institute of Homeopathy will meet at Buffalo June 24th, 1897, and continue in session for the usual time. On the 23d the Materia Medica Conference will convene and hold three sessions, two on Wednesday, and one on Thursday morning. The new society of Ophthalmologists will also be in session on Wednesday, and there is no doubt of a large attendance at the opening of the Institute.

The programme of the Institute has already been made out in outline and may contain some novel features. But this much may now be said: That the sectional chairmen have nearly all arranged definite and clear-cut programmes; will furnish a *few* fine papers instead of the usual hit or miss lot, and will have carefully arranged discussions, and in many cases will furnish abstracts of the papers. This reform in itself would almost revolutionize matters, and all that can be accomplished in this direction in one year will be done.

The attractions of Buffalo, the beauty and power of Niagara must not be overlooked. The local committees are and have been hard at work, and those who know predict great things as the result of their labors. Let every member of the Institute bring or send one new member, and we will add three hundred new names to the roll at Buffalo.

EUGENE H. PORTER, M. D.
*General Secretary.*

Institute meeting, June 24th, 1897, Buffalo, New York.

